# A Neuron-Specific Deletion of the MicroRNA-Processing Enzyme DICER Induces Severe but Transient Obesity in Mice

**DOI:** 10.1371/journal.pone.0116760

**Published:** 2015-01-28

**Authors:** Géraldine M. Mang, Sylvain Pradervand, Ngoc-Hien Du, Alaaddin Bulak Arpat, Frédéric Preitner, Leonore Wigger, David Gatfield, Paul Franken

**Affiliations:** 1 Center for Integrative Genomics, University of Lausanne, Lausanne, Switzerland; 2 Mouse Metabolic Evaluation Facility, Center for Integrative Genomics, University of Lausanne, Lausanne, Switzerland; 3 Genomic Technologies Facility, Center for Integrative Genomics, University of Lausanne, Lausanne, Switzerland; 4 Vital-IT, SIB-Swiss Institute of Bioinformatics, Lausanne, Switzerland; The University of Tokyo, JAPAN

## Abstract

MicroRNAs (miRNAs) are small, non-coding RNA molecules that regulate gene expression post-transcriptionally. MiRNAs are implicated in various biological processes associated with obesity, including adipocyte differentiation and lipid metabolism. We used a neuronal-specific inhibition of miRNA maturation in adult mice to study the consequences of miRNA loss on obesity development. *Camk2a-CreERT2* (*Cre^+^*) and floxed *Dicer* (*Dicer^lox/lox^*) mice were crossed to generate tamoxifen-inducible conditional *Dicer* knockouts (cKO). Vehicle- and/or tamoxifen-injected *Cre^+^;Dicer^lox/lox^* and *Cre^+^;Dicer^+/+^* served as controls. Four cohorts were used to a) measure body composition, b) follow food intake and body weight dynamics, c) evaluate basal metabolism and effects of food deprivation, and d) assess the brain transcriptome consequences of miRNA loss. cKO mice developed severe obesity and gained 18 g extra weight over the 5 weeks following tamoxifen injection, mainly due to increased fat mass. This phenotype was highly reproducible and observed in all 38 cKO mice recorded and in none of the controls, excluding possible effects of tamoxifen or the non-induced transgene. Development of obesity was concomitant with hyperphagia, increased food efficiency, and decreased activity. Surprisingly, after reaching maximum body weight, obese cKO mice spontaneously started losing weight as rapidly as it was gained. Weight loss was accompanied by lowered O_2_-consumption and respiratory-exchange ratio. Brain transcriptome analyses in obese mice identified several obesity-related pathways (e.g. leptin, somatostatin, and nemo-like kinase signaling), as well as genes involved in feeding and appetite (e.g. *Pmch, Neurotensin*) and in metabolism (e.g. *Bmp4*, *Bmp7*, *Ptger1*, *Cox7a1*). A gene cluster with anti-correlated expression in the cerebral cortex of post-obese compared to obese mice was enriched for synaptic plasticity pathways. While other studies have identified a role for miRNAs in obesity, we here present a unique model that allows for the study of processes involved in reversing obesity. Moreover, our study identified the cortex as a brain area important for body weight homeostasis.

## Introduction

MicroRNAs (miRNAs) are small, non-coding RNA molecules that act as post-transcriptional repressors of gene expression. They promote mRNA decay and inhibit translation by base pairing to the 3′ untranslated regions of target transcripts. In mammals, they may target as much as 50% of all protein-coding mRNAs [1]. MiRNAs are involved in most cellular processes investigated thus far [2] and their dysregulation contributes to many human diseases [3,4]. Functional studies implicate miRNAs in the regulation of metabolism and in metabolic disorders related to cholesterol and lipid metabolism, adipocyte differentiation, insulin secretion, and glucose homeostasis [5,6]. MiRNAs therefore gain growing attention in the field of diabetes and obesity research.

In vertebrates, the generation of mature miRNAs critically depends on the RNase-III enzyme DICER [7]. In the central nervous system, the cell type-specific deletion of *Dicer* has revealed essential roles for miRNAs in neural differentiation and proliferation, as well as in brain development and cognitive function [8–11]. To circumvent the neurodevelopmental problems associated with *Dicer* deficiency [12], we used an inducible, neuron-specific conditional knockout system based on *Camk2a*-driven CreERT2, to delete *Dicer* in adult mice using tamoxifen [13]. This strategy has previously been shown to cause a down-regulation of miRNAs in *Camk2a*-expressing cells [10]. We observed that the neuron-specific deletion of *Dicer* rapidly induced severe and highly reproducible obesity. Importantly, the obesity phenotype was transient and spontaneously reversed to join the normal growth curve of untreated littermate controls. The development of obesity was associated with transcriptional changes in the cerebral cortex, affecting several obesity-related pathways and the control of feeding behavior.

## Methods

### Animals and housing conditions

All mice used in this study were individually housed in polycarbonate cages (31 × 18 × 18 cm) in a sound-attenuated and temperature/humidity-controlled room (25°C, 50–60% respectively). Mice were kept under a 12h light/ 12h dark cycle (lights on at 9 am, 70–90 lux) and had access to food and water *ad libitum. Camk2a-CreERT2* and *Dicer^lox/lox^* mice were purchased from Jackson laboratory (Bar Habor, ME, USA; Stock Number 012362 and 006366, respectively). All experiments were approved by the Ethical Committee of the State of Vaud Veterinary Office, Switzerland.

### Generation of *Dicer* conditional knockout (cKO) mice

*Dicer* cKO (*Cre^+^/Dicer^lox/lox^*) were obtained by crossing hemizygous *Camk2a-CreERT2* (*Cre^+^*) animals with homozygous *Dicer* floxed (*Dicer^lox/lox^*) mice. Both lines were created on a mixed 129/SvxC57BL/6 and maintained on a C57BL/6J background by backcrossing to C57BL/6J animals for at least 9 generations. The *Camk2a-CreERT2* transgenic mouse line expresses a tamoxifen-activable Cre-recombinase under the control of the brain-specific *Camk2a* (calcium/calmodulin-dependent protein kinase II alpha) promoter [14]. The *Dicer* conditional allele contains loxP sites on either side of exon 24 of the *Dicer1 gene,* an exon encoding most of the second RNase III domain. Cre-mediated recombination results in the removal of 90 amino-acids of the protein [15]. Genetic recombination was induced in adult mice by treatment with the exogenous estrogen-receptor ligand tamoxifen, according to Metzger and Chambon [13]. Tamoxifen was purchased from Sigma-Aldrich, Buchs, Switzerland (Product N°: T-5648) and dissolved in a sunflower seed oil/ethanol mixture (10:1) at a final concentration of 10mg/ml. The suspension was sonicated for 10 minutes to obtain a homogeneous solution. 8-week-old animals were injected intraperitoneally with 1mg of tamoxifen (100μl) twice a day over five consecutive days. Each mouse thus received a total of 10mg of tamoxifen. Control animals were injected with 100μl of sunflower seed oil/ethanol mixture (vehicle) at the same frequency. In all experiments, vehicle-injected *Cre^+^;Dicer^lox/lox^* animals served as controls. For the metabolic assay, tamoxifen- and vehicle-injected *Cre^+^/Dicer^+/+^* mice were used as additional controls, to exclude a possible effect of tamoxifen itself, or of the non-induced *Cre*-transgene.

### Genotyping *Cre^+^* and *Dicer^lox/lox^* mice

**Figure 1:**
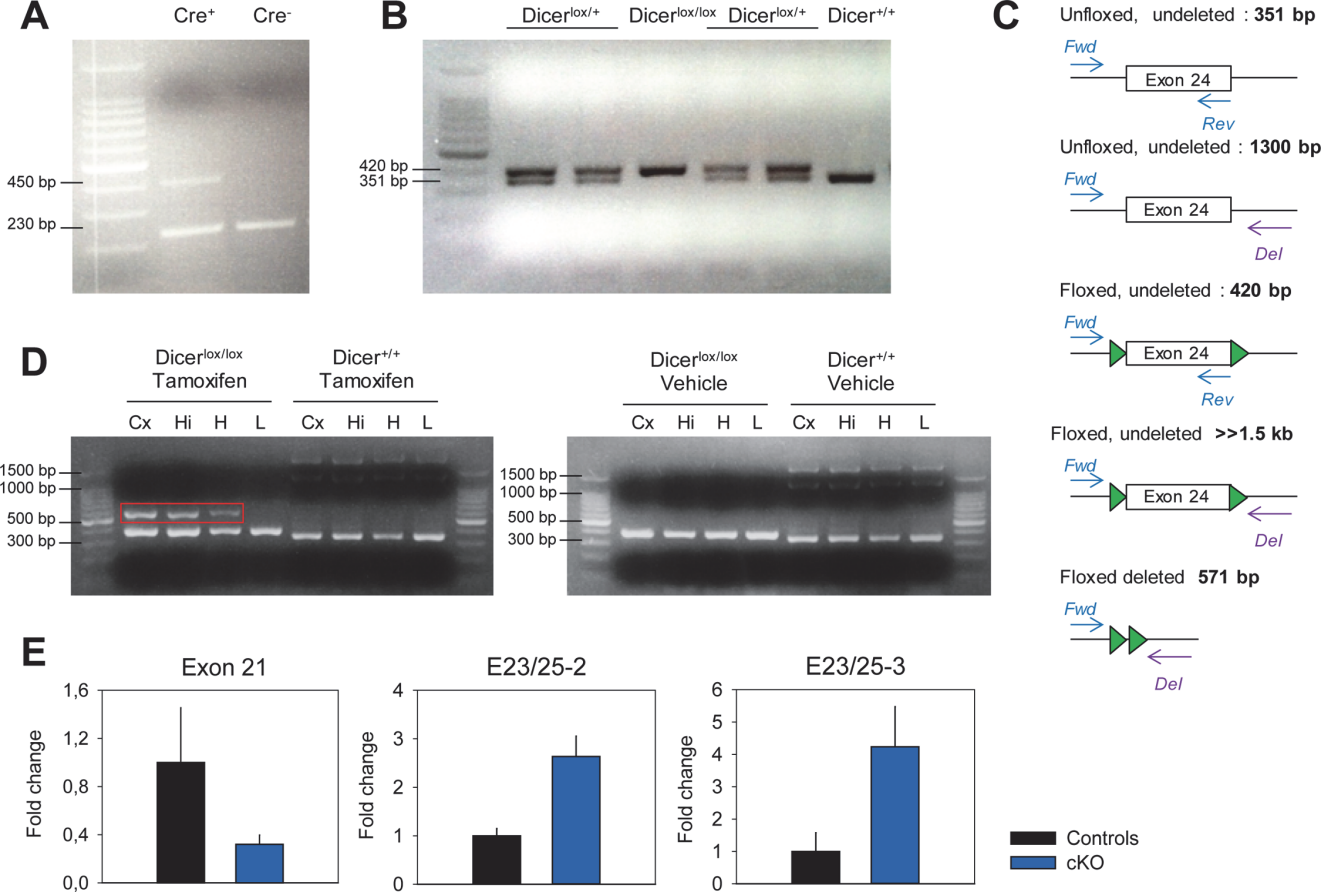
Verification of the genetic recombination in *Dicer* conditional knockout (cKO) mice. A. PCR of genomic DNA to test for the presence (left lane; *Cre^+^*; 450 bp PCR product) or absence (right lane; *Cre^−^*; 230 bp control band) of the *Cre* transgene. **B and C**. PCR of *Dicer* mice to test the presence of loxP sites surrounding exon 24 of *Dicer*. The wild type allele resulted in a 351 bp band, whereas the presence of loxP sites resulted in a 420 bp PCR product, as expected. **C and D**. Injecting *Cre^+^;Dicer^lox/lox^* mice with tamoxifen led to the Cre-mediated excision of exon 24. PCRs were carried out on three different brain tissues i.e., cortex (Cx) hippocampus (Hi), hypothalamus (H), with three primers (i.e. Forward (Fwd) and Reverse (Rev) and Forward and Deleted (Del), see C. Only the brain samples from tamoxifen-induced *Dicer^lox/lox^* mice showed the 571 bp band that is diagnostic of productive recombination at the *Dicer* locus (red box in left panel of D). The large band of 1300 bp in the unfloxed mice (*Dicer^+/+^*) originated from primers Forward and Deleted on the wild-type allele. Note that recombination occurred only in the brain and not in liver (L). Also note that the *Dicer^lox/lox^* mice injected with tamoxifen still showed the non-recombined band (420 bp), indicating that not all cells in the tissue recombined, as expected from the presence of *Camk2a*-negative neurons and non-neuronal cells. **E.** Tamoxifen injection in *Cre^+^;Dicer^lox/lox^* mice (n = 2/genotype) resulted in a down-regulation of *Dicer* mRNA in the hippocampus, indicated by the decrease in exon 21 expression, and in an increase in the splicing of exon 23 onto exon 25. Expression of the exon23–25 splice variant was quantified using two different primer pairs; i.e., E23/25−2 and -3. Exon 21 expression was normalized to that of 3 reference genes, the expression of the exon23-25 splice variant to that of exon 21. Control values were set to 1.

Mouse ear-punch samples were used for genotyping the presence of the *Cre* transgene and the floxed *Dicer* alleles (Fig. 1A and B). DNA was extracted and purified using the Maxwell 16 Tissue DNA Purification Kit (Promega AG, Dübendorf, Switzerland) according to the manufacturer’s instructions. 2.5 μl of the supernatant was used for Polymerase Chain Reaction (PCR) amplification with specific primers for the *Cre* transgene (Forward: 5′AGGTGTAGAGAAGGCACTTAGC3′ and Reverse: 5′CTAATCGCCATCTTCCAGCAGG3′) and a positive control (Forward: 5′CCAATCCCTTGGTTCATGGTTGC3′ and Reverse: 5′CGTAAGGCCCAAGGAAGTCCTGC3′) and the *Dicer* floxed and deleted allele (Forward: 5′CCTGACAGTGACGGTCCAAAG3′, Reverse: 5′CATGACTCTTCAACTCAAACT3′, Deleted: 5′GGGCAGCCCCATCTCAAAGGCCTACCTGAG3′). PCR products were loaded on 2% agarose gels to check the presence of the *Cre* transgene (450 bp), and its positive control (230 bp), the wild type *Dicer* allele (351 bp), the *Dicer* floxed allele (420 bp), and the deleted allele (≈571 bp; Fig. 1).

### Verification of the recombination by RT-PCR

cDNA was synthesized from 1μg of total RNA from the hippocampus of cKO and control mice, using random hexamers and SuperScript II reverse transcriptase (Invitrogen, Carlsbad, CA, USA) according to manufacturer’s instructions. cDNA was PCR-amplified using FastStart Universal SYBR Green Master mix (Roche, Basel, Switzerland). To quantify the appearance of exon23-25 spliced transcript upon knockout of exon 24, we used two different pairs of primers targeting the junction between exon 23 and 25: E23/25−2 (Forward: 5′ACGCCTCCTACCACTACAAC3′ and Reverse: 5′TTTCTCCTCATCCTCCTCGG3′) and E23/25∂3 (Forward: 5′AACACTATCACTGCTTAGGAGAT3′ and Reverse: 5′TGGGGACTTCGATATCCTCTTC3′). Expression of the skipped exon 23–25 was normalized to that of exon 21, taken as an internal reference (Forward: 5′GAGTCACCTGCTAAGCTCCA3′ and Reverse: 5′ACATTCATTGCTGGGCTTGG3′). Exon 21 expression was quantified to assess the overall down-regulation of *Dicer* mRNA, and normalized to three reference genes: *Eef1a1* (Forward: 5′ TCCACTTGGTCGCTTTGCT3′ and Reverse: 5′CTTCTTGTCCACAGCTTTGATGA3′), *Nudt4* (Forward: 5′ AAGTTCAAGCCCAACCAGACG3′ and Reverse: 5′TCCTGGGACAATCCATTGGTC3′), and *Trip12* (Forward: 5′ TCCCTGGGATCTACAACACCA3′ and Reverse: 5′ATCATCTCTGCTATGCTGCAAG3′). The mean expression level for each exon in cKO samples was expressed relative to that of the control samples (n = 2/genotype).

### Body weight and composition

Three cKO and 3 control mice were used to assess body weight changes over 90 days, starting the day of the first injection (day 0). Fat and lean body mass were determined on isoflurane-anesthetized animals using a whole-body composition analyzer (EchoMRI LLC, Houston, TX, USA) on day 65.

### Food intake

Four cKO and 4 control mice were used to follow food intake and body weight dynamics. Starting at the day of tamoxifen/vehicle injection, body weight and food consumption were measured every 2–3 days over 17 weeks. Food efficiency was calculated on a weekly basis, as follows:

$${\text{Food efficiency}}_{\left[t\right]}=(\left({\text{body weight}}_{\left[t\right]}-{\text{body weight}}_{\left[t-1\right]})/({\text{food intake}}_{\left[t\right]}-{\text{food intake}}_{\left[t-1\right]}\right))*100$$

where [*t*] and [*t*-1] indicate consecutive weeks.

### Metabolism

Four experimental groups were used to analyze metabolic parameters: cKO mice (n = 6), control mice (n = 6), and *Cre^+^/Dicer^+/+^* injected with tamoxifen or vehicle (n = 6 and 5, respectively). Indirect calorimetry was performed using the Oxymax system (Columbus Instruments, Columbus, OH, USA). Metabolic rate was analyzed over the course of a ‘fed-fasted-refed’ protocol, in which mice were fed *ad lib* for 72h (i.e., 3 consecutive 24h days starting 2.5h before dark onset). On day 3, food was removed 2.5h before dark onset for 15h, and for the subsequent 24h mice were refed *ad lib*. Oxygen consumption (VO_2_) and carbon dioxide production (VCO_2_) were measured to calculate energy expenditure (heat production) and the respiratory exchange ratio (RER). Due to a technical problem of the calorimeter, food intake could not be reliably measured during the metabolic experiments. Mice were recorded twice; 21 and 42 days after tamoxifen/vehicle treatment with the first recording occurring at the beginning of the weight gain phase and the second recording immediately after body weight peaked; i.e., at the beginning of the decreasing phase.

### Statistical analysis

cKO and control groups were compared using unpaired, 2-tailed Student’s t-tests. Time courses were analyzed by 2-way repeated measures (RM) ANOVA with factors ‘Time’ and ‘Treatment’; LSD tests were used as post-hoc analysis. Significance threshold was set at p = 0.05.

### Gene expression

Twelve cKO and 12 control mice were used for analyses of gene expression in different brain regions. Transcriptome analyses were performed at two time points; 4 weeks after tamoxifen/vehicle treatment, when mice were already obese but did not reach their peak body weight, and 8 weeks after treatment when the mice were losing weight and returning to normal body weight. Animals were sacrificed 3–4 h after light onset and cerebral cortex, hypothalamus, and hippocampus were dissected using a microscope as follows: the hypothalamus was extracted from the ventral side of the brain, as described in [16]; resulting in a sample measuring 2mm from either side of the third ventricle laterally, from optic chiasm to the posterior border of the mammillary bodies, and the thalamus dorsally. For cortex and hippocampus dissection, two 2mm-thick sagittal slices taken 1mm lateral from the inter-hemispheric fissure were cut using a sagittal slicer matrix (Alto Stainless Matrices, Stoelting CO, IL, USA) in PBS (Life Technologies Europe, Zug, Switzerland). From the two resulting slices, hippocampi and cortex were dissected according to the Mouse Brain Atlas [17]. Brain tissues were homogenized in QIAzol Lysis Reagent (Qiagen, Hombrechtikon, Switzerland). Total RNA was extracted and purified using the RNeasy Lipid Tissue Mini Kit 50 (Qiagen, Hombrechtikon, Switzerland) according to manufacturer’s instructions. All RNA sample amounts were measured with a NanoDrop ND-1000 spectrophotometer (Thermo Scientific, Wilmington, NC, USA) and the quality of RNA samples was verified on Agilent 2100 bioanalyzer chips (Agilent technologies, Basel, Switzerland). Total RNA (100ng) was used to perform target preparation using Ambion WT Expression Kit (Life Technologies Europe, Zug, Switzerland). 5.5μg of each fragmented cDNA was end-labeled with biotin and hybridized to a Mouse 430.2.0 Microarray (Affymetrix, Santa Clara, CA, USA) according to manufacturer’s instructions. For each brain region, tissues from 3 animals per treatment and time point were used, resulting in a total of 12 arrays/tissue (36 arrays total). Microarrays were processed and scanned according to standard procedures. Expression data were normalized and summarized with the robust multi-average (RMA) method implemented in the Affymetrix Expression Console. In addition, the MAS5 normalization algorithm was run to obtain a detection call for each probe set on each array. These algorithms were applied separately to the data from each tissue. Probe sets not called present in at least 3 samples and unannotated probe sets (Affymetrix annotation na33) were removed resulting in 14′713 probe sets in cortex, 14′527 in hippocampus, and 15′460 in hypothalamus. For each gene we kept the probe set with the highest variance across the 12 arrays from a tissue. Limma was used to fit a linear model to the expression data with the four conditions as factors [18]. The comparisons between tamoxifen and vehicle treated animals at each time point were extracted using contrast matrices. *P* values were adjusted for multiple testing with Benjamini and Hochberg’s method to control the false discovery rate (FDR) [19]. Since the expression of many genes in the cortex was altered at 4 weeks with a low fold-change, we applied a non-stringent FDR cut-off of 15%. GeneGo Software (MetaCore, Thomson Reuters, New York, NY, USA) was used for biological analysis of the enriched pathways and cellular processes between cKO and control animals in the microarray data. Raw data are available on GEO database (Accession Number GSE61937).

## Results

### Tamoxifen injection induces a brain-specific deletion of *Dicer* exon 24

Tamoxifen injection in adult mice induced the proper excision of *Dicer* exon 24 in *Cre^+^;Dicer^lox/lox^* mice (cKO mice) but not in *Cre^+^;Dicer^+/+^* control mice (Fig. 1). Recombination in cKO occurred only in the three brain regions tested; i.e., cortex, hippocampus, and hypothalamus, but not in the liver, and only brain samples from tamoxifen-injected *Dicer^lox/lox^* mice showed the 571 bp band that is diagnostic of the recombination at the *Dicer* locus (red box in left panel of D). Vehicle-injected *Cre^+^;Dicer^lox/lox^* and tamoxifen-injected *Cre^+^;Dicer^+/+^* did not show any recombination, in none of the tissues tested (Fig. 1D). The large band of 1300 bp in the unfloxed mice (*Dicer^+/+^*) originated from primers Forward and Deleted on the wild-type allele. However, cKO mice still showed the non-recombined band (420 bp), indicating that not in all cells recombination occurred, as expected from the presence of non-*Camk2a*-expressing neurons and other cells (e.g. glia). We reasoned that in the absence of exon 24, there would be an increased splicing of exon 23 onto exon 25, which could serve as an independent and quantifiable molecular marker for knockout efficiency. Indeed, in the hippocampus of cKO animals, we found a significant increase in exon23-25 splicing (Fig. 1E). Moreover, the level of *Dicer* mRNA seemed down-regulated in the hippocampus of cKO mice, as exemplified by the reduction in exon 21 (Fig. 1E), consistent with the results reported by Konopka *et al.* [10].

### The neuronal knock-down of *Dicer* induces severe but transient obesity, associated with hyperphagia and altered food efficiency

**Figure 2:**
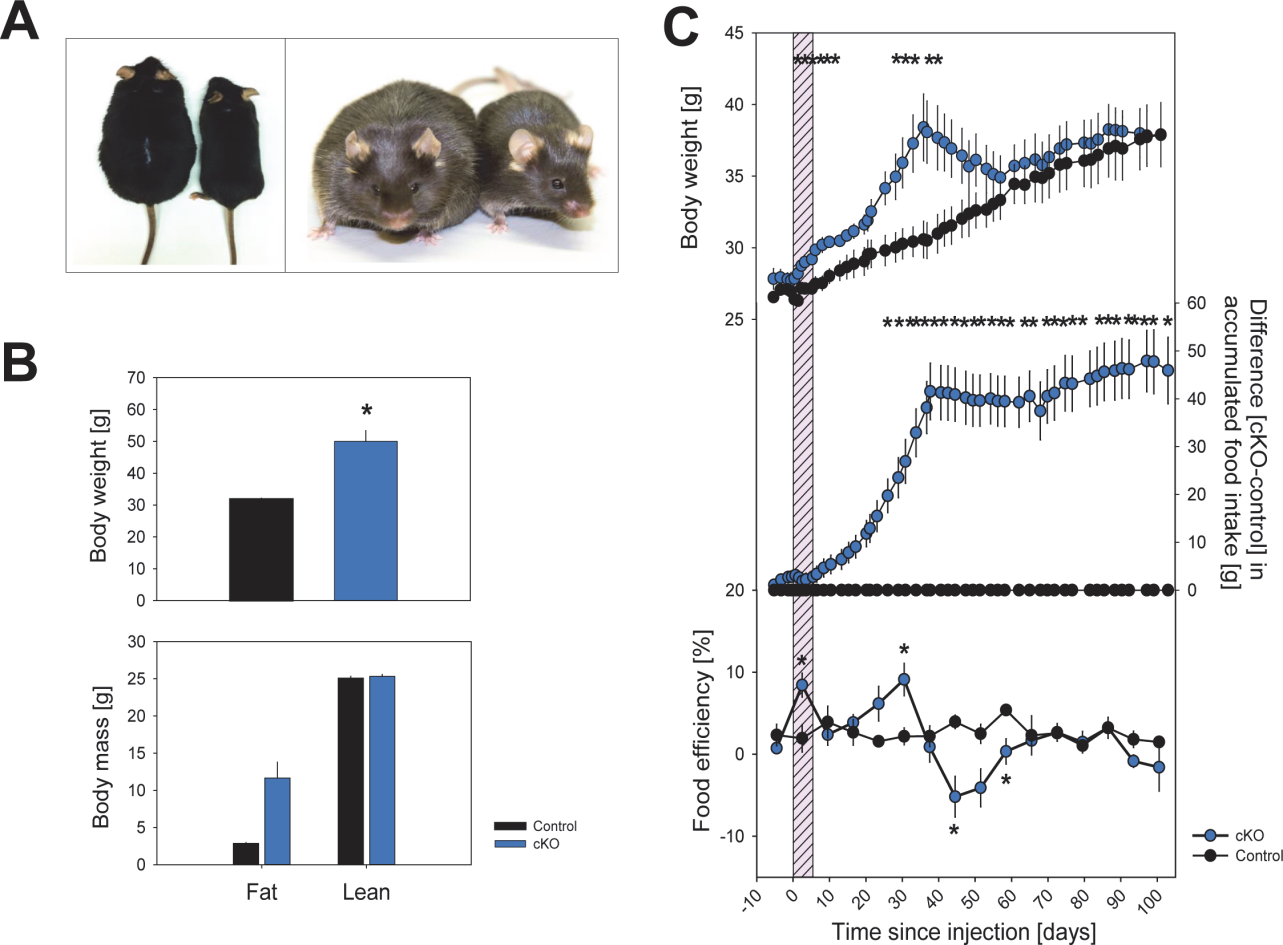
*Dicer* cKO mice show transient obesity. A.Photographs of two representative *Cre^+^;Dicer^lox/lox^* mice injected either with tamoxifen (cKO, 55.2 gr) or vehicle (27.2 gr) taken 49 days after injection. The same two mice are represented from a dorsal (left picture) and frontal (right picture) view. **B.** *Upper panel.* Body weight of cKO and control mice at day 49 (n = 3/group). *Lower panel.* Fat and lean body mass of cKO and control mice at day 65 (n = 3/group). Note that at this time, body weight in cKO mice already decreased. **C.** Body weight and food intake in cKO mice and controls (n = 4/group) over a 17-week period. Dashed grey panel highlights the 5 days of tamoxifen treatment. cKO mice increased their body weight concomitant with increasing food intake until reaching a maximum at day 38. Values reverted to control levels within 3 weeks. Food efficiency was calculated per week. Data expressed as mean (±1 SEM). cKO and control mice are represented in blue and black, respectively. Stars mark significant differences (t-tests p<0.05).

The brain-specific deletion of *Dicer* led to the rapid development of obesity, in all cKO animals we tested thus far (n = 38 in total). In a first cohort, cKO mice accumulated 18.5 g extra weight (or +59%), 47.5 days after tamoxifen injection compared to controls (Fig. 2A and 2B). This increase in body weight was mainly due to an increase in fat mass (Fig. 2B). The time at which body weight peaked did, however, differ among the experimental groups in which the exact peak time was determined [experiment 1 (body composition): day 47.5±1.5, n = 3; experiment 2 (food efficiency): day 38.5±0.5, n = 4; experiment 3 (indirect calorimetry): day 42.0 ±0.0, n = 6; ANOVA factor ‘cohort’: p<0.01; all 3 experiments: day 42.2±0.9, n = 13]. Weight gain at peak time did not significantly differ among cohorts (+12.4±2.2 g or +39%, n = 13 cKO vs. 26 controls). The increase in body weight was associated with increased food intake and food efficiency (Fig. 2C and S1 Fig.), suggesting that obesity was not solely due to increased food intake, but that both hyperphagia and reduced metabolism contributed to the rapid accumulation of white adipose tissue.

After reaching maximum body weight, cKO mice spontaneously entered a fast descending phase in which they lost weight as rapidly as it was gained (Fig. 2C). The switch from gain to loss occurred within a very short time window and was highly reproducible in that in each of the 38 cKO mouse from 4 independent experimental cohorts showed the same pattern of body weight changes after recombination. At the switch from weight gain to weight loss, cKO mice entered a catabolic state in which food intake abruptly changed from strong hyperphagia to mild hypophagia documented in Fig. 2C by the steep positive slope in cumulative feeding between days 18–39 (i.e., the linear phase of increase: +1.6 gr/day, R^2^ = 0.99, P<0.001) to the slight but significant negative slope between days 39–70 (−0.1 gr/day, R^2^ = 0.62, P<0.001; linear regression on mean values). This mild hypophagia is, however, not sufficient to explain the pronounced and fast decrease in body weight. This discrepancy is compellingly illustrated by the large negative food efficiency values observed during the catabolic state that dropped from +9.1 during obesity to −5.2%, while in control mice food efficiency remained constant at an intermediate +2.6% throughout the experiment (Fig. 2C). Finally and similarly surprising was the observation that after losing the extra weight they gained, cKO mice joined the normal growth curve of the control mice (Fig. 2C). These data strongly suggest that both altered feeding and disturbed metabolism contributed to this unique obesity phenotype.

### Obesity is associated with reduced basal metabolism, reduced locomotor activity, and unresponsiveness to fasting

To further investigate this metabolic aspect, indirect calorimetry was used to assess oxygen consumption (VO_2_), heat production, and locomotion at two time points. A first, 5-day recording was carried out starting 21 days after tamoxifen injection; i.e., the time food efficiency started increasing in cKO mice just before entering the ascending phase of body weight gain (referred to as “pre-obese”). This 5-day experiment was repeated on day 42 after tamoxifen injection; the time window when mice reached peak body weight (referred to as “obese”). cKO mice were compared to three control groups: *Cre^+^;Dicer^+/+^* mice injected with tamoxifen and *Cre^+^;Dicer^+/+^* and *Cre^+^;Dicer^lox/lox^* mice injected with vehicle. No difference was observed among the three control groups (p>0.1, see S2 Fig.) and values were averaged for presentation. The lack of a difference among controls demonstrated that the effects observed were not due to tamoxifen or to the *Cre*-transgene.

**Figure 3:**
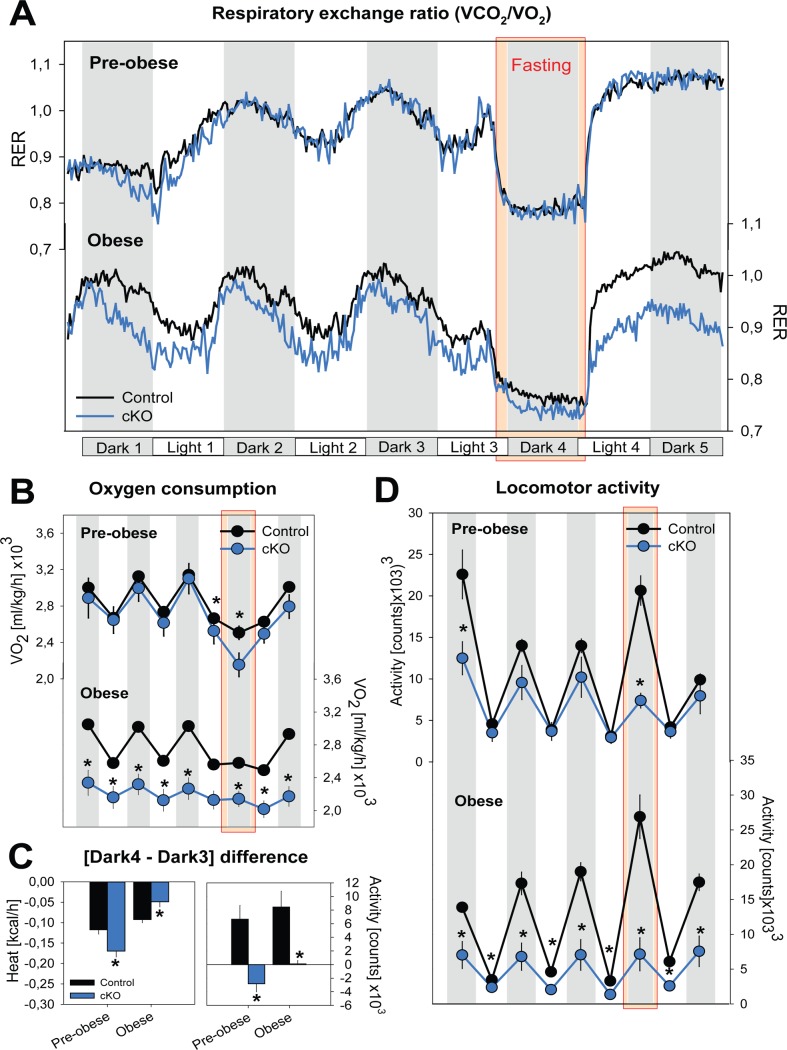
*Dicer* cKO mice show reduced basal metabolism and activity, and an altered response to fasting. Metabolic parameters and locomotor activity were measured during an *ad lib* fed state (72h baseline) followed by a 15h overnight fasting period and subsequent 24h refeeding. Data are shown as mean (±1 SEM) and statistical differences are indicated by stars above graphs (t-tests, p<0.05). Values of the three control groups (see text) were averaged. **A and B**. Compared to control mice (black; n = 18), obese cKO mice (blue; n = 6) showed reduced respiratory exchange ratio both under baseline and in response to fasting, mostly due to a lower VO_2_. Pre-obese mice show only a reduction in VO_2_ during fasting. **C.** Both pre-obese and obese cKO mice fail to enhance locomotor activity and showed a larger and smaller drop in heat production, respectively. **D**. Obese cKO mice have an overall reduction of locomotor activity, both in baseline and in response to fasting.

During baseline, pre-obese cKO mice had normal respiratory exchange ratio (RER) and VO_2_ values, but were less active (Fig. 3; ANOVA, factors ‘Time’ and ‘Group’: p<0.001). Obese cKO mice showed a lower baseline metabolism, as evidenced by a lower VO_2_ (Fig. 3A and B) and a lower RER (preferential lipid oxidation), consistent with a catabolic state. In addition, obese cKO mice, like the pre-obese cKO mice, were less active than control mice (Fig. 3D).

To evaluate metabolic adaptation to changes in energy supply, mice were challenged with a 15h period of fasting. In control animals, fasting induced a decrease in metabolism (i.e., reduced VO_2_ and heat production, Fig. 3B and 3C), a metabolic switch to lipids (decrease in RER; Fig. 3A), and increased locomotor activity, likely reflecting food seeking behavior [Fig. 3D [20]]. In pre-obese cKO mice, fasting induced a larger drop in both VO_2_ and heat production as compared to controls (Fig. 3B and C). Conversely, in obese cKO mice, fasting only marginally reduced VO_2_ and heat production compared to controls (Fig. 3B and C) likely as a result of the baseline state being already catabolic, consistent with the lower RER in both fed and refed states. Interestingly, cKO mice were also less responsive to fasting in terms of enhancing locomotor activity (Fig. 3C and D). This lack of food-seeking behavior did not depend on body weight and was observed in both the pre-obese and obese states (analysis not shown). Together, these data indicate that neuronal deletion of *Dicer* disrupted metabolic balance and impaired behavioral and metabolic adaptation to rapid changes in energy supply.

### The brain transcriptome of obesity dynamics

**Figure 4:**
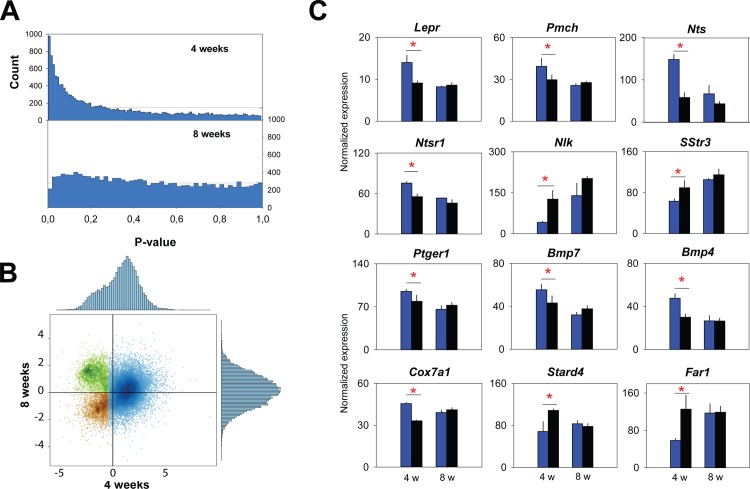
Gene expression of *Dicer* cKO and control mice in the cortex. A.P-value histograms for [cKO-control] comparisons in the cortex at 4 and 8 weeks. The horizontal line represents the number of P values expected by chance. The continuous decrease in P values from 0 to 0.4 suggests that a majority of genes are differentially expressed at 4 weeks. At 8 weeks none of the transcripts were differentially expressed. **B.** Scatter plot and distributions of the moderated t-statistic at 4 weeks and 8 weeks for 14′713 genes in the cortex. X- and Y-axis: moderated t from the cKO-control contrast at 4 and 8 weeks, respectively. At 4 weeks, the t-statistic distribution is skewed with a majority of genes having positive values. At 8 weeks, the distribution is symmetrical with a modal value close to zero. The three clusters (brown, green, blue) were defined by fitting a two-component Gaussian mixture model on each of the two marginal distributions (“mclust” R). Brown: data belonging to component 1 in both distributions. Green: data belonging to component 1 in the 4 weeks distribution and to component 2 in the 8 weeks distribution. Blue: data belonging to the 4 weeks component 1 which did not separate into two distinct clusters at 8 weeks. **C**. Mean (±1 SEM) expression of genes related to food intake, thermogenesis, and lipid metabolism (n = 3/condition). Blue bars represent the cKO mice and black bars the controls. Bars are grouped by 4-week (4 w, left pair of bars) and 8-week (8 w, right bar pair) values.

Although conditional *Dicer* deletion has been shown to importantly decrease most miRNAs in the targeted cells [10,12,21,22], to gain insight into the specific molecular pathways that could underlie the obesity phenotype, we investigated the changes in mRNA expression in the brain. We performed cortical, hypothalamic, and hippocampal transcriptome analyses, in a fourth cohort 4 weeks after treatment, when mice showed body weight gain (+8.1±1.1 g or +28.3%, n = 6 compared to controls; t-test p<0.001), and 8 weeks after treatment, when body weight no longer differed from control levels (+4.8±2.4 g or +14.9%; t-test p = 0.11). At 4 weeks, we observed a large number of transcripts that were significantly affected by the absence of *Dicer* specifically in the cerebral cortex (Fig. 4 and S3 Fig.). For both the 4- and 8-week time points, no differences could be observed in the hypothalamus, neither in the hippocampus (S3 Fig.). This lack of response was not due to a larger variability among biological replicates in these two tissues compared to cortex (S4 Fig.).

**Figure 5:**
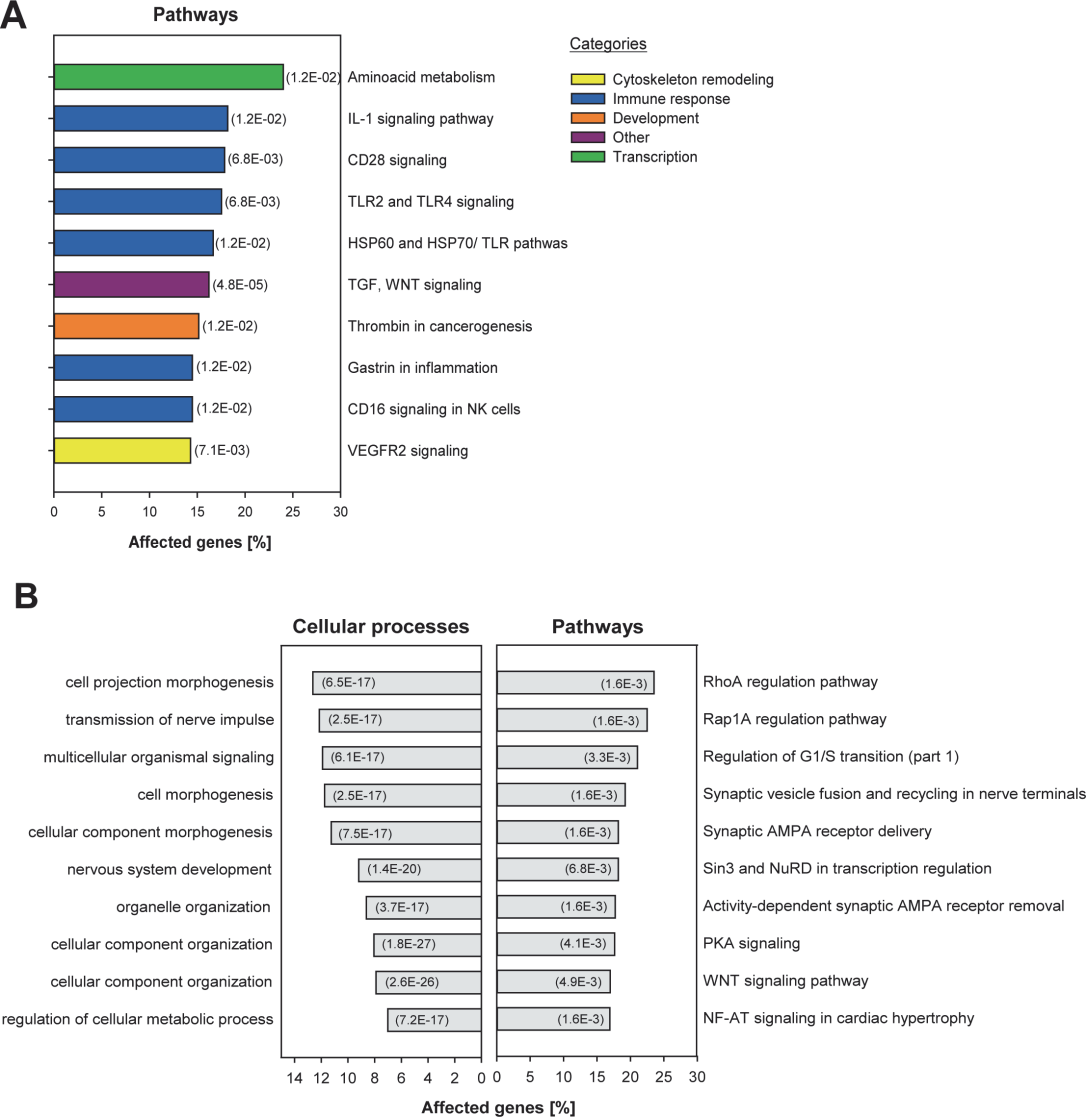
Pathway analysis of significant genes at 4 weeks and of the anti-correlated cluster at 8 weeks. **A.** Top 10 enriched pathways in cKO mice at 4 weeks. The % genes affected within each pathway are represented. The bar colors indicate categories to which the pathway belongs. **B.** Top 10 enriched cellular processes (left) and pathways (right) found in the cluster of anti-correlated genes (i.e., the green cluster in Fig. 4B).

In the cortex, 1020 genes were differentially expressed in cKO mice 4 weeks after treatment. Of those, 828 (i.e., 81%) were up-regulated (Fig. 4A, S1 Table). Several among those have documented links to feeding control and maintaining proper energy balance; e.g., the leptin receptor (*Lpr*) and pro-melanin-concentrating hormone (*Pmch*) were up-regulated, as were neurotensin (*Nts*) and its receptor (*Ntsr1*; Fig. 4A and C, S1 Table). Expression of other genes, such as Nemo-like kinase (*Nlk*) and somatostatin receptor 3 (*Sstr3*) were down-regulated in cKO mice and thus are unlikely direct miRNA targets. Interestingly genes involved in heat production through white-to-brown adipocyte transition, such as prostaglandine E receptor 1 (*Ptger1*), cytochrome c oxidase (*Cox7a1*) and bone morphogenetic proteins (*Bmp4-7*) were also found up-regulated in the cortex of cKO mice (Fig. 4C, S1 Table). Other genes that participate in lipid metabolism (*Dgkd, Far1, Pld1, Stard4*) and immune-related responses (*Hsp90aa1, Hspb8, Il22, Il4*) showed significantly changed expression as well (Fig. 4C, S1 Table). Among the top 10 gene-ontology pathways for this group of 1020 affected transcripts, we found an enrichment for several immune response and inflammation-related pathways (Fig. 5A).

At 8 weeks, no genes were found differentially expressed in the cortex of cKO and control mice. Nevertheless, a specific cluster of genes showed an anti-correlated effect between the two time points (Fig. 4B, S2 Table) and which therefore might be involved in restoring metabolic balance. Pathway and cellular processes enrichment analysis on the 1094 genes belonging to this cluster showed that enriched pathways were linked to synaptic plasticity and AMPA receptors in particular, whereas enriched cellular processes were mostly associated with cell morphogenesis and cellular component organization (Fig. 5B).

## Discussion

Here, we described the use of a transgenic approach to investigate the consequences of adult neuronal miRNA loss on body weight, feeding behavior, and metabolism in adult mice. The time- and brain-specific deletion of *Dicer* in adult mice occurred only in a sub-population of cells, mostly post-mitotic neurons of hippocampal and cortical origin. Although *Camk2a*-expressing neurons are abundantly present in these two areas [10,14], *Camk2a*-negative neurons and non-neuronal cells are thus expected to contribute to the presence of non-recombined alleles. Future experiments using different approaches (e.g. FACS sorting neurons based on *Camk2a*-expression and/or determining the cell-type specificity of the recombination by *in situ* hybridization for *Dicer* and/or for specific miRNAs) could help to define the precise molecular mechanisms underpinning the phenotype. In the recombined cells, the excision of exon 24 resulted in lower levels of *Dicer* mRNA, consistent with previous reports [10, 21]. This decrease in *Dicer* mRNA is probably due to a lower stability of the mutant transcript because pre-mRNA levels were not altered [21].

The neuronal-deletion of the miRNA-processing enzyme DICER led to the development of rapid and transient obesity in mice. Surprisingly, this phenotype has not been described by others using a very similar *Dicer* cKO strategy in adult mice, neither in mice lacking *Dicer* in neurons from birth (i.e., non-inducible *Camk2a-Cre^+^; Dicer^lox/lox^* mice) [10,23]; but see statement below). In the latter model, whole brain transcriptome analysis showed an up-regulation of transcripts involved in regulating gene expression through chromatin modifications, whereas down-regulated genes were associated with neuronal function and axogenesis [23]. The same study found that although many miRNAs were affected, no particular enrichment was found for their targets among the genes for which mRNA levels changes were found [23], illustrating the difficulty of identifying specific molecules using genome-wide approaches.

In *Dicer* cKO mice, the weight gain phase was associated with hyperphagia, increased food efficiency and reduced metabolism. To what extent hyperphagia is cause or consequence of metabolic dysfunction is, however, unclear. The hyperphagia, reduced metabolism, and increased fat mass of our cKO mice is comparable to other mouse models of obesity such as leptin- and leptin-receptor-deficient mice [24,25], that develop obesity from early age, and a maturity-onset mouse obese model in which the melanocortin system was targeted [26]. However, in none of these models a spontaneous reversal of obesity was observed, which is, to our knowledge, unique to our *Dicer* cKO mice.

Several miRNAs have been found to be causally involved in obesity [6]. The specific deletion of the *miR-143/145* cluster protected mice from developing dietary-induced obesity [27]. Similarly, overexpression of *miR-802* led to the development of obesity-associated insulin resistance [28]. Interestingly, inhibition of *miR-200a* could reverse obesity by increasing the level of the leptin receptor and insulin receptor 2 in the hypothalamus [29]. Conversely, in the hippocampus, leptin was recently shown to decrease the expression of several genes directly targeted by *miR-132*, suggesting that leptin can drive the expression of this particular miRNA [30]. These and other miRNAs could thus be regarded as novel targets in the treatment of obesity and related metabolic disorders. Whether any of the aforementioned miRNAs was responsible for the obesity phenotype observed in our cKO mice is difficult to assess as deletion of *Dicer* affects virtually all miRNAs.

Gene expression analysis revealed that most (i.e., 81%) of the affected genes in the cortex were up-regulated in cKO mice, consistent with an absence of miRNA’s repressor activity. At 4 weeks, several obesity-related genes and genes involved in the control of feeding behavior were significantly affected in cKO mice; *Lpr* and *Pmch* were up-regulated, as were *Nts* and its receptor *Ntsr1*. These genes are known to be crucial for maintaining proper energy balance and *Lpr* has been previously shown to be under the control of *miR-200*a [29,31,32]. Moreover, both *Pmch* and *Nts* were recently shown to be part of the motivational and hedonic aspect of feeding behavior [31,32]. Other genes, such as *Nlk, which* was recently found as part of potential susceptibility loci for obesity [33], and *Sstr3 [34]* were also changed in cKO mice. Besides the control of food intake, also metabolic pathways were deregulated in the cortex of cKO mice. In particular, several genes involved in thermogenesis through brown adipose tissue and in the white-to-brown adipocyte transition were affected (e.g., *Cox7a1, Bmp4, Bmp7, Ptger1*) [35–37], as well as a whole set of genes related to lipid metabolism and transport (e.g. *Far1, Dgkg, Stard4*). Together, these data support the notion of miRNAs being involved in the control of adipogenesis, lipid metabolism and adaptive thermogenesis.

The brain transcriptome analysis also revealed an enrichment of transcripts that are part of inflammatory pathways and immune response at 4 weeks, which could have contributed to the development of the phenotype. Whether inflammation is a cause or a consequence of obesity is, however, unclear. Chronic inflammation in the circulation and peripheral metabolic tissues is one of the hallmarks of obesity [38]. Moreover, recent evidence shows crucial roles for neuroinflammatory processes in metabolic disorders [39], especially in the hypothalamus in which inflammatory responses could lead to a dysregulation of feeding [40]. These observations indicate that, besides the specific transcripts known to be causally linked to feeding behavior (see above), a variety of pathways indirectly associated with energy metabolism were affected in cKO mice. The dysregulation of neuronal miRNAs thus had wide-ranging and complex effects on gene expression which likely contributed to the development of the obesity phenotype.

The most interesting observation in our model mice is probably the spontaneous reversal of obesity. This surprising phenotype indicates that after deletion of miRNAs, compensatory mechanisms take place to re-establish neuronal networks and these modifications might underlie the recovery of normal energy homeostasis. Our observations of enrichment in synaptic plasticity-related pathways are consistent with the remodeling of synapses and changes in dendritic morphology that has been reported in a similar construct of *Camk2a*-specific *Dicer* deletion [10].

As an alternative explanation to specific miRNAs and their mRNA targets underlying the dysregulation and subsequent recalibration of metabolic homeostasis, we could surmise that neuronal loss and subsequent replacement could underlie our unique obesity phenotype. Two recent studies have used *Dicer* knockout strategy to delete POMC-neurons in the hypothalamus, and reported the development of obesity in parallel to cell death within the first 6 weeks of age [41,42]. This metabolic phenotype was associated with changes in transcript levels in the hypothalamus for genes known to be linked to neurodegeneration supporting the idea that obesity resulted from the loss of specific cells in the brain. The return to normal body weight regulation that we observe would require restoration of neuronal function through e.g. neurogenesis and/or establishment of new neuronal connections. Although research on adult neurogenesis has focused mainly on the hippocampus and the subventricular zone of the lateral ventricle, recent studies reported the existence of neurogenesis in other brain areas, notably in the hypothalamus and in the cortex, where it has functional implication in the control of feeding behavior [43]. In the cortex of our cKO mice, as stated earlier, several inflammatory pathways and immune response genes were enriched at 4 weeks, and neuro-inflammatory processes are known to trigger neurogenesis [44,45]. Moreover, the time course of weight gain and loss in the cKO mice fits the time course of adult neurogenesis in rodents, taking place within 4–7 weeks [46]. Further investigation of cell death and adult neurogenesis in *Dicer* cKO mice might help to test this hypothesis.

Contrary to expectation, no differences in gene expression were observed between tamoxifen and vehicle treated *Dicer* cKO mice in the hypothalamus, a brain region well known for its role in the control of food intake and energy homeostasis [47], nor in the hippocampus, a region for which there is growing evidence for an involvement in metabolic control [48]. One factor that could have contributed to the negative results in the hypothalamus is this tissue’s higher heterogeneity and transcriptome analyses targeting specific nuclei could be informative. The fact that many transcripts were affected in *Dicer* knockout mice were observed in the cortex suggest a role for cortical areas in feeding behavior especially in the motivational aspect of feeding [48,49].

## Conclusion

We have discovered a unique and robust mouse model of obesity in which both the metabolic dysregulation leading to obesity as well as the homeostatic compensatory processes that correct this dysregulation can be studied within a short time window. Identifying the processes that are implicated in the return to normal body weight will bring a new perspective in the treatment of obesity and metabolic disorders.

While this manuscript was under review, Vinnikov *et al.*, similarly reported on transient obesity in inducible, *Camk2a*-driven, *Dicer* KO mice [50]. They confirmed the development of hyperphagic obesity associated with increased fat mass and a body weight dynamics similar to that observed in our study, underscoring the robustness and high reproducibility of our results. Using different techniques, the authors also confirmed that the recombination occurred in several brain regions, including the cerebral cortex, hippocampus, and hypothalamus. Obesity in their mouse model was associated with increased circulating leptin levels, and an increase in the orexigenic factor NPY in the hypothalamus, an observation that is consistent with our microarray data suggesting a deregulation of food intake control in the brain. Vinnikov *et al*, could identify one miRNA, *miR-103*, targeting the PI3K/Akt/mToR pathway, as a key component of the initial development of obesity. The authors further noted that the deletion of *Dicer* induces neurodegeneration in hippocampus specifically, an observation which is in line with our hypothesis outlined above suggesting that neurodegenerative processes followed by neurogenesis and associated synaptic plasticity changes and reorganization of cellular networks could underlie restoration of body weight homeostasis. Together, the two complementary studies provide mechanistic insight both into the development of body weight gain, as well as into the reversal of obesity.

## Supporting Information

S1 Fig Food intake accumulation in cKO and control mice. *Upper panel.* Cumulated food intake (mean ± 1 SEM) in cKO mice and their controls (n = 4/group) over a 17-week period. *Lower panel.* cKO-control differences (mean +/− 1 SEM) in accumulation of food intake. Starting after injection of tamoxifen, cKO mice increased food intake until reaching a maximum at day 38. Values reverted to control levels within 3 weeks and subsequently followed the normal growth curve with stable food intake. cKO and control mice are represented by dark and light blue dots, respectively. Black dots represent the cKO-control differences. Stars above the graphs correspond to significant results of post-hoc student’s t-tests p<0.05.
(TIF)

S2 Fig Metabolic parameters in the three control groups and in cKO mice. Data are shown as mean (± SEM). The three control groups (i.e., *Cre^+^;Dicer^lox/lox^* injected with vehicle, purple lines and dots, n = 6, *Cre^+^;Dicer^+/+^* with vehicle, blue lines and dots, n = 5, *Cre^+^;Dicer^+/+^* with tamoxifen, green lines and dots, n = 6) and the cKO group (red lines and dots, n = 6) are shown. No difference was observed between the three control groups, neither at the pre-obese, nor at obese time point. **C.** Note that although in all 3 control groups activity increased during fasting (night 4) there was a significant difference among groups in the degree by which locomotor activity was increased (Dark4-Dark3 difference). For details see legend Fig. 3.
(TIF)

S3 Fig P value histograms for the tamoxifen vs. vehicle comparisons in the three brain regions at 4 and 8 weeks. P values were computed using R package “limma” (see methods). The horizontal dashed line represents the number of P values expected by chance. For details see legend Fig. 4.
(TIF)

S4 Fig Coefficient of variation (CV) within each experimental group. The CV was calculated as the ratio of the standard deviation divided by the mean of the three biological replicates for each condition in each tissue. The hippocampus and hypothalamus, that do not show any difference between tamoxifen and vehicle conditions, do not have a larger within group variability.
(TIF)

S1 Table List of transcripts that are significantly changed between cKO and control mice in the cortex at 4 weeks. (XLSX)

S2 Table List of transcripts with anti-correlated expression between 4 and 8 weeks in the cortex. (XLS)
