# Supplementary figures and images for: A Neuron-Specific Deletion of the MicroRNA-Processing Enzyme DICER Induces Severe but Transient Obesity in Mice
Source: PLoS One. 2015 Jan 28;10(1):e0116760. doi: 10.1371/journal.pone.0116760 (PMC4309537; doi:10.1371/journal.pone.0116760)

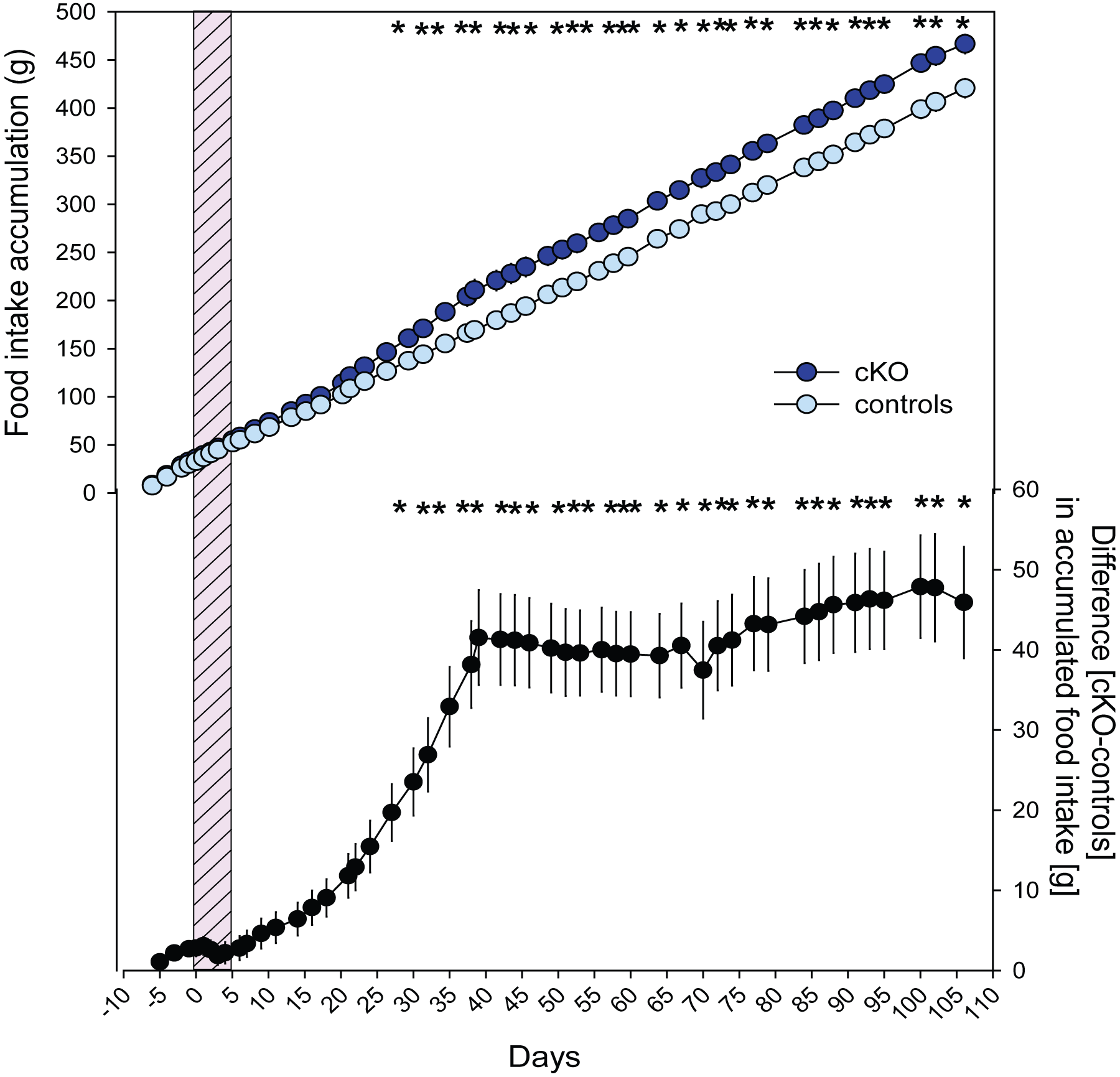

Supplement: S1 Fig — Upper panel. Cumulated food intake (mean ± 1 SEM) in cKO mice and their controls (n = 4/group) over a 17-week period. Lower panel. cKO-control differences (mean +/− 1 SEM) in accumulation of food intake. Starting after injection of tamoxifen, cKO mice increased food intake until reaching a maximum at day 38. Values reverted to control levels within 3 weeks and subsequently followed the normal growth curve with stable food intake. cKO and control mice are represented by dark and light blue dots, respectively. Black dots represent the cKO-control differences. Stars above the graphs correspond to significant results of post-hoc student’s t-tests p<0.05. (TIF) [file pone.0116760.s001.tif]

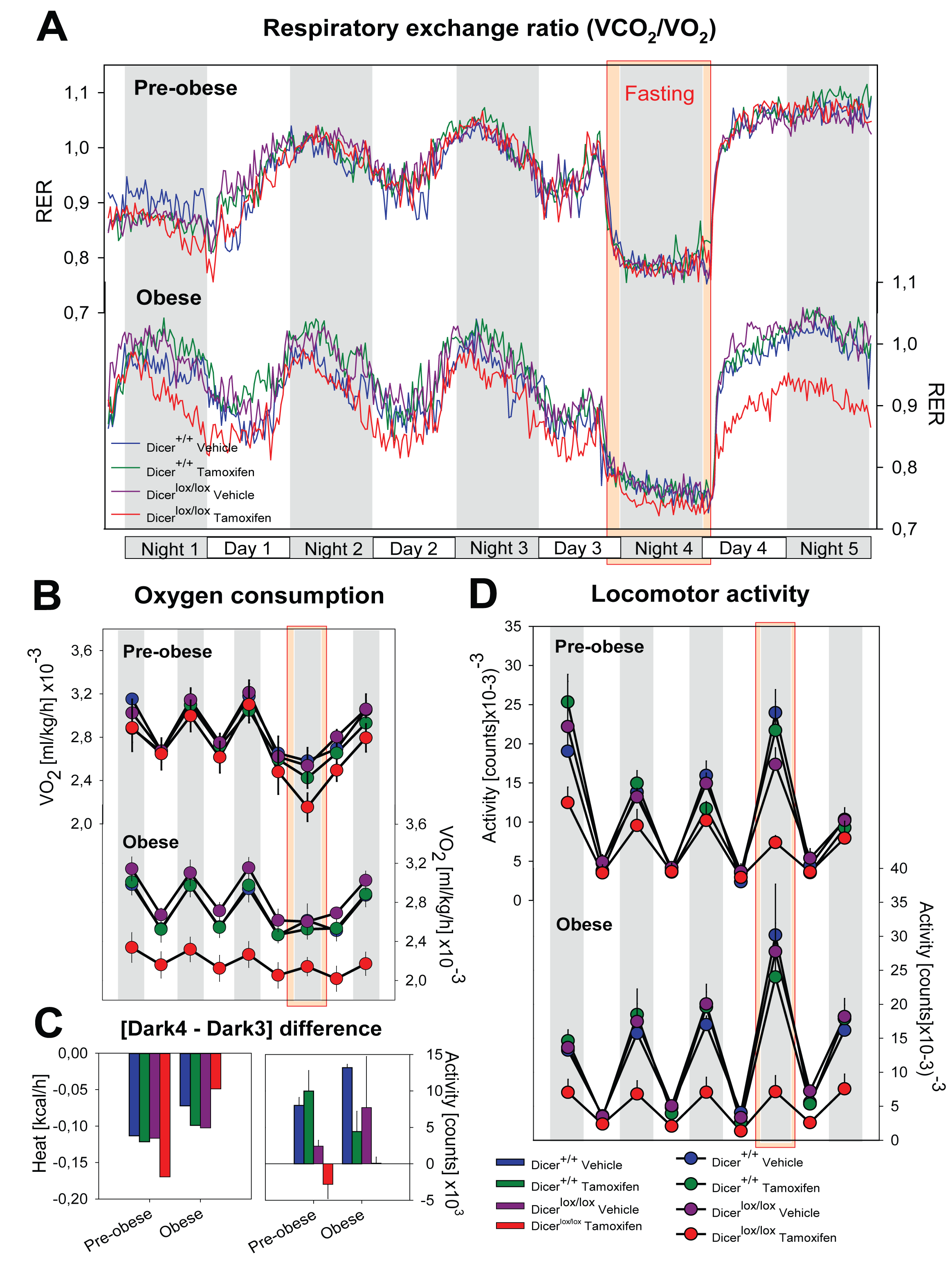

Supplement: S2 Fig — Data are shown as mean (± SEM). The three control groups (i.e., Cre+;Dicerlox/lox injected with vehicle, purple lines and dots, n = 6, Cre+;Dicer+/+ with vehicle, blue lines and dots, n = 5, Cre+;Dicer+/+ with tamoxifen, green lines and dots, n = 6) and the cKO group (red lines and dots, n = 6) are shown. No difference was observed between the three control groups, neither at the pre-obese, nor at obese time point. C. Note that although in all 3 control groups activity increased during fasting (night 4) there was a significant difference among groups in the degree by which locomotor activity was increased (Dark4-Dark3 difference). For details see legend Fig. 3. (TIF) [file pone.0116760.s002.tif]

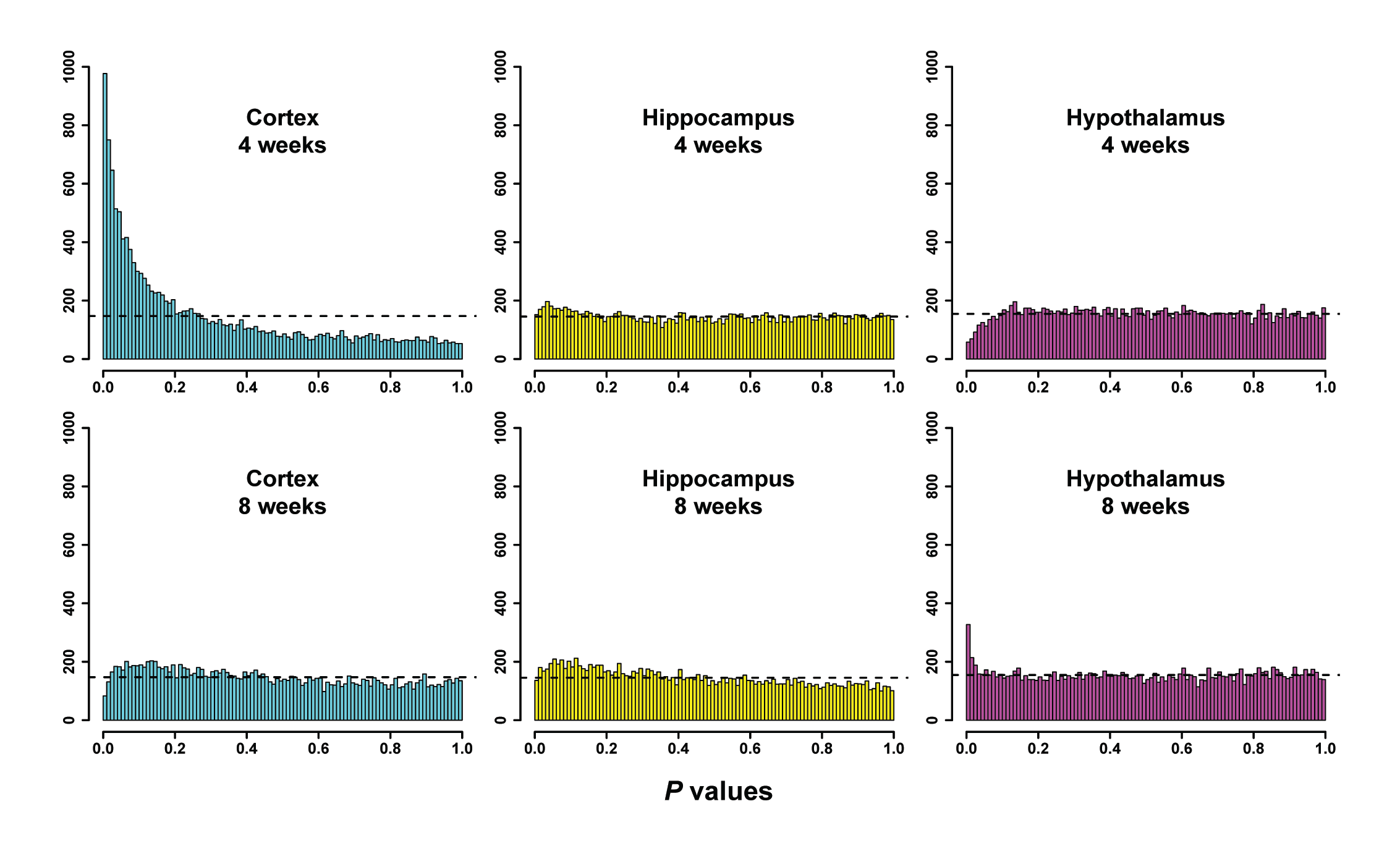

Supplement: S3 Fig — P values were computed using R package “limma” (see methods). The horizontal dashed line represents the number of P values expected by chance. For details see legend Fig. 4. (TIF) [file pone.0116760.s003.tif]

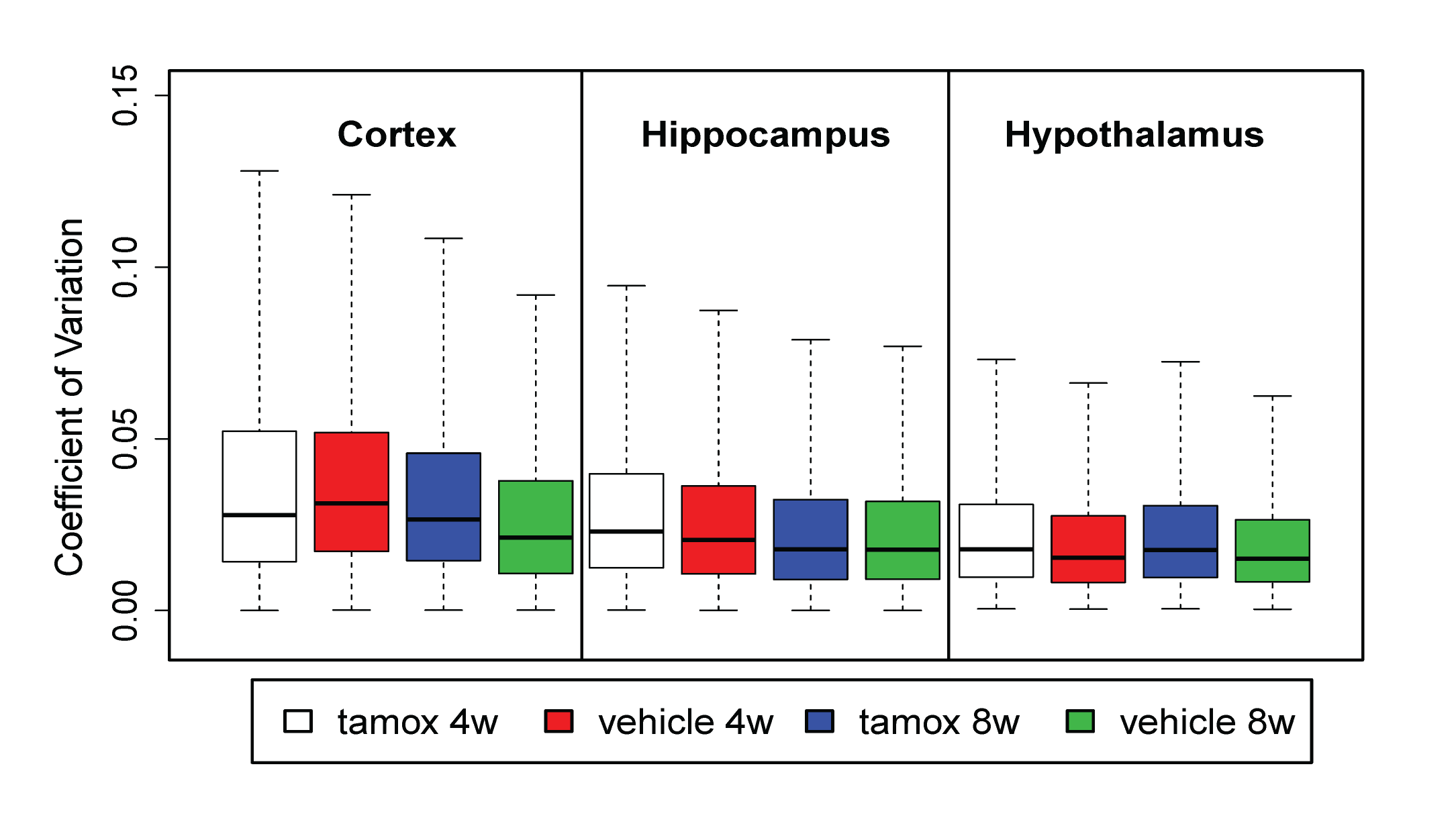

Supplement: S4 Fig — The CV was calculated as the ratio of the standard deviation divided by the mean of the three biological replicates for each condition in each tissue. The hippocampus and hypothalamus, that do not show any difference between tamoxifen and vehicle conditions, do not have a larger within group variability. (TIF) [file pone.0116760.s004.tif]
